# Sexual risk behaviors among adolescent men who have sex with men in two Brazilian cities: findings from an RDS-based study

**DOI:** 10.1590/1980-549720260033

**Published:** 2026-09-11

**Authors:** Marto Leal, Carl Kendall, Rosa Livia Freitas de Almeida, Antonio José Lima de Araujo, Carlos Erasmo Sanhueza Sanzana, Cristiane Cunha Frota, Jennifer Glick, Paula Meireles, Henrique Barros, Tatiana Monteiro Fiuza, Telma Alves Martins, Alexandre Grangeiro, Laio Magno, Ines Dourado, George Rutherford, Artur Olhovetchi Kalichman, Ivana Cristina de Holanda Cunha Barreto, Maria Clara Gianna, Maria Mercedes Escuder, Mirleide de Brito Figueiredo, William McFarland, Marcos Cavalcante Paiva, Aline Rodrigues Feitoza, Lígia Kerr

**Affiliations:** I Universidade Federal do Ceará, Department of Community Health - Fortaleza (CE), Brazil.; II Tulane University, Department of Behavioral, Social and Population Sciences - New Orleans (Louisiana), United States of America.; III Universidade Federal do Ceará, Department of Pathology and Forensic Medicine - Fortaleza (CE), Brazil.; IV Louisiana State University, Health Sciences Center - New Orleans (Louisiana), United States of America.; V Universidade do Porto, Public Health Institute - Porto, Porto, Portugal.; VI Secretaria da Saúde do Estado do Ceará - Fortaleza (CE), Brazil.; VII Universidade Estadual Paulista “Júlio de Mesquita Filho” - São Paulo (SP), Brazil.; VIII Universidade do Estado da Bahia, Department of Life Sciences - Salvador (BA), Brazil.; IX Universidade Federal da Bahia, Health Collective Institute - Salvador (BA), Brazil.; X University of California, Department of Epidemiology and Biostatistics - San Francisco (San Francisco), United States of America.; XI Secretaria Municipal de Saúde - São Paulo (SP), Brazil.; XII Fiocruz - Eusébio (CE), Brazil.; XIII Instituto de Saúde - São Paulo (SP), Brazil.; XIV Secretaria de Saúde de Fortaleza, Fortaleza, Ceará, Brazil.

**Keywords:** Sexual behavior, HIV, Men who have sex with men, Adolescence, Comportamento sexual, HIV, Homens que fazem sexo com homens, Adolescência

## Abstract

**Objective:**

To estimate the prevalence of sexual risk behaviors among adolescent men who have sex with men and to identify factors associated with these behaviors.

**Methods:**

Data were collected between December 2020 and July 2022 in Fortaleza and São Paulo, Brazil, using respondent-driven sampling. Study outcomes included inconsistent condom use, multiple sexual partners, and sexualized drug use.

**Results:**

The final sample was composed of 287 adolescents. Among participants, 38.0% reported inconsistent condom use, 36.1% reported multiple sexual partners, and 23.8% reported sexualized drug use. Factors associated with inconsistent condom use were not having a steady partner (adjusted prevalence ratio (APR): 0.5), having come out to everyone or almost everyone (APR: 6.3), consuming alcohol weekly (APR: 2.9), looking for family/friends/pharmacy (APR: 0.7), or looking for the public healthcare service when sick (APR: 0.7), and having sexual intercourse with partners of unknown HIV status (APR: 1.6). Factors associated with multiple sexual partners were receiving money in exchange for sex (APR: 1.6), and having sexual intercourse with partners of unknown HIV status (APR: 2.0). Factors associated with sexualized drug use included Catholic religion (APR: 0.2), having a steady partner < 20 years old (APR: 2.0), participating in non-governmental organization activities (APR: 2.5), experiences of violence (APR: 2.7), receiving money in exchange for sex (APR: 1.7), and having sexual intercourse with partners of unknown HIV status (APR: 2.1).

**Conclusion:**

These findings highlight the high prevalence of sexual risk behaviors among adolescent men who have sex with men, underscoring the need for targeted prevention strategies and youth-friendly services in diverse urban contexts.

## RESUMO

**Objetivo:**

Estimar as prevalências de comportamentos sexuais de risco entre adolescentes homens que fazem sexo com homens e identificar os fatores associados a esses comportamentos.

**Métodos:**

Os dados foram coletados entre dezembro de 2020 e julho de 2022, em Fortaleza e São Paulo, Brasil, utilizando respondent-driven sampling. Os desfechos do estudo incluíram uso inconsistente de preservativo, múltiplos parceiros sexuais e uso de drogas sexualizadas.

**Resultados:**

A amostra final foi composta de 287 adolescentes. Entre os participantes, 38% relataram uso inconsistente de preservativo; 36,1%, múltiplos parceiros sexuais; e 23,8%, uso de drogas sexualizadas. Os fatores associados ao uso inconsistente de preservativo foram: não ter parceiro fixo (APR: 0,5), estar assumido para todas ou quase todas as pessoas (APR: 6,3), uso semanal de álcool (APR: 2,9), buscar cuidado com familiares/amigos/farmácia (APR: 0,7) ou em serviço público de saúde (APR: 0,7) quando adoecido, e ter relações sexuais com parceiros de status sorológico desconhecido (APR: 1,6). Os fatores associados aos múltiplos parceiros sexuais foram: receber dinheiro em troca de sexo (APR: 1,6) e ter relações sexuais com parceiros de status sorológico desconhecido (APR: 2,0). Os fatores associados ao uso de drogas sexualizadas incluíram: religião católica (APR: 0,2), ter parceiro fixo com menos de 20 anos (APR: 2,0), participação em atividades de ONG (APR: 2,5), experiências de violência (APR: 2,7), receber dinheiro por sexo (APR: 1,7) e relações sexuais com parceiros de status sorológico desconhecido (APR: 2,1).

**Conclusão:**

Identificou-se alta prevalência de comportamentos sexuais de risco entre adolescentes homens que fazem sexo com homens, ressaltando a necessidade de estratégias de prevenção de serviços acolhedores para jovens em diferentes contextos urbanos.

## INTRODUCTION

Men who have sex with men (MSM) are 26 times more likely to acquire HIV than the general population^1^. This group has experienced the largest proportional increase in new HIV infections worldwide over the past decade, representing the greatest growth observed among all key populations^2^. In 2019, MSM represented almost 25% of all new HIV infections worldwide^1^. In Brazil, the HIV prevalence among MSM increased 50% between 2009 and 2016; primarily among low-income and young MSM^3^.

Adolescent men who have sex with men (AMSM) are a critical group to prioritize in HIV prevention, given a myriad of additional risk factors. As Mayer et al. point out in their comprehensive review article, AMSM have additional risks for HIV because their impulse control is less developed and they are less familiar with serostatus and other risk mitigation discussions. AMSM are among the few populations with increasing numbers of new HIV infections in parts of the world where HIV incidence is decreasing^4^ ^,^ ^5^. This group is at higher risk of HIV infection compared to young heterosexual men^6^ and older MSM^4^. Data from the United States estimated that 80% of new HIV infections occur among AMSM^7^.

Key HIV sexual risk behaviors among adolescents are inconsistent condom use (ICU) during sex; sexualized drug use (SDU); and multiple sexual partners (MSP)^8^. Demographic, behavioral, and social factors can be associated with these behaviors^9^ ^,^ ^10^ ^,^ ^11^, including the age of the partners^12^, socioeconomic status^13^, supportive parent-child relationships^14^, and educational aspects^15^.

This study aimed to estimate the prevalence of sexual risk behaviors among AMSM in two Brazilian cities and identify factors associated with these behaviors.

## METHODS

### Study design, population and sample

This is a cross-sectional study conducted between December 2020 and July 2022. The study population consisted of AMSM aged 15-19 years in two Brazilian capitals, Fortaleza, representing the Brazilian northeast, and São Paulo, the largest city in Brazil, representing the southeast region.

These cities were selected because they represent contrasting socioeconomic and cultural contexts in Brazil, illustrating regional disparities that influence HIV vulnerability among adolescents. The cities, and their respective regions, vary greatly on the Human Development Index, ranging from 0,842 in São Paulo to 0,745 in Fortaleza^16^. Additionally, both cities host long-standing research groups with experience in respondent-driven sampling (RDS) and previous HIV surveillance studies among MSM, including the 12-city national survey^17^. This existing expertise and infrastructure facilitated recruitment, community engagement, and the implementation of standardized procedures across sites.

Male adolescents between 15 and 19 years old, cisgender, who lived and/or studied and/or worked in one of the participating cities, and who reported having at least one sexual intercourse (anal/oral) with men in the previous 12 months were included in the study.

The AMSM population sample size was calculated using the percentage of MSM in each city (2.0% in Fortaleza and 4.7% in São Paulo)^18^. We applied a HIV prevalence of 4.6% and 3.6% among MSM aged 18 to 19 years old in Fortaleza and São Paulo, respectively^17^, a confidence interval of 95%, and an acceptable error of 2.5%. We calculated a sample size of 239 for Fortaleza and 211 for São Paulo.

### Recruitment and data collection

Participants were recruited using RDS. RDS was originally developed by Heckathorn and Broadhead^19^ ^,^ ^20^ ^,^ ^21^ and has become widely used for surveillance in hard-to-reach populations that cannot be accessed conventionally^22^. RDS is a recruitment and analysis method, and both components are necessary for its use.

The first subjects recruited, also known as “seeds”, were selected non-randomly during the formative research carried out with ten AMSM in Fortaleza and seven in São Paulo. Seeds were selected for their popularity, for having large social networks, and for representing diverse ethnicities, socioeconomic status, and educational strata.

Each seed received three unique recruitment coupons and was given instructions on what information to provide to their friends/recruits. AMSM who arrived at the study site with a valid coupon and who met the other inclusion criteria constituted the first “wave” of the study. The potential participant was supposed to hand in their coupon to the field supervisor, complete the eligibility questions, and receive an explanation of the study’s objectives, procedures, and risks, as well as the benefits of participating. Those who were eligible and who agreed to participate signed a consent form and were enrolled in the project. Individuals who were ineligible or who declined participation were thanked for their time and provided with educational material about HIV prevention and condoms.

Eligible participants were then directed to a private room, where a trained professional administered the questionnaire using a virtual form, ensuring information confidentiality and privacy^23^.

After the interview, participants received three new coupons, linked to their original coupon, to invite their eligible friends. Each participant who attended the site and participated in the study received a primary incentive of R$ 25.00 (~US$ 5.40) in Fortaleza and R$70.00 (~US$15.20) in São Paulo, and a secondary incentive of the same value for each subject invited who attended and participated in the study. Incentive amounts differed between the two cities because of the relative cost of living.

### Measures

ICU, MSP, and SDU were identified as study outcomes because they are considered the main sexual risk behaviors for HIV among adolescents.

ICU was defined as not using a condom in every anal sex intercourse (whether as a inserter or a received) with a steady and/or casual male partner; MSP was defined as sexual intercourse with two or more sexual partners in the last three months, simultaneously or not; and SDU was defined as using a combination of two variables, namely participation in sex parties where drugs were used, and report of alcohol and/or other drugs (marijuana, heroin, cocaine, amphetamine, LSD, and ecstasy) interfering in the use of condoms during sexual intercourse.

Independent variables included: sociodemographic characteristics (age, race); social relationships; experience of physical, psychological, and/or sexual violence; sexual behaviors; use of HIV preventive methods; participation in non-governmental organizations (NGO) activities; and use and abuse of alcohol and other drugs. We applied standardized questions commonly used in epidemiological surveys with MSM^17^.

Each outcome was analyzed separately as a dependent variable. Outcomes were not used as independent variables in models of other outcomes to simplify interpretation and avoid redundancy.

### Statistical analysis

Analyses were conducted in several steps to account for the RDS design and for methodological challenges associated with self-reported network size among AMSM. First, we addressed potential heaping, under-reporting, and over-reporting of personal network size (degree), as well as missing or implausible values. Instead of using the raw self-reported degree, we applied the visibility-imputation model proposed by McLaughlin et al.^24^, which combines each participant’s reported degree, the number of recruits they brought into the study, and the time to recruitment to estimate a smoothed “visibility” value. The objective of this step was to reduce the influence of extreme or inconsistent degree reports and to obtain more plausible degree estimates for weighting.

Second, using these imputed visibilities as degrees, we calculated individual RDS weights with the Gile successive sampling estimator in RDSAnalyst. This estimator is appropriate when the target population is finite and the sampling fraction is non-negligible, as in our study, and its objective is to correct for unequal selection probabilities inherent to RDS recruitment.

Third, we imported these individual weights into Stata^®^ 17 and specified a complex survey design, treating each city (Fortaleza and São Paulo) as a separate stratum. All descriptive and regression analyses incorporated the RDS weights and the stratification by city. We then estimated weighted prevalences and corresponding 95% confidence intervals (95% CI) for the main variables of interest.

Fourth, we examined crude associations between each outcome (ICU, MSP, and SDU) and the explanatory variables using weighted Pearson’s chi-square tests.

Finally, for each outcome, we fitted a separate multivariable Poisson regression model with robust variance to estimate adjusted prevalence ratios (APR) and 95% CI. Three p-value thresholds were used: p<0.20 to select variables for inclusion in the multivariable model, p<0.05 to retain variables in the final model, and p<0.01 to highlight statistically stronger associations in descriptive comparisons. All multivariable Poisson regression models were adjusted for age and for all covariates presenting p<0.20 in the crude analyses for each respective outcome.

The overall objective of this analytic strategy was to preserve the advantages of RDS for accessing a hard-to-reach population while minimizing bias introduced by degree misreporting, differential recruitment, and unequal selection probabilities.

### Ethical Approval

The study was approved by the Universidade Federal do Ceará Ethics Committee (No. 4345875), which granted ethical approval for all study procedures conducted at both research sites, Fortaleza and São Paulo. All participants signed an informed consent form. For adolescents aged 15-17 years, a judicial waiver authorized them to provide their own consent without parental or guardian permission because such authorization could expose those who had not disclosed their sexual orientation and potentially place them in situations of risk or distress.

## Data Availability Statement:

The dataset supporting the results of this study is not publicly available.

## RESULTS

The final sample was composed of 287 adolescents, 76 in Fortaleza and 211 in São Paulo. The median age of AMSM was 17 years (interquartile range (IQR):17-18).

Among all participants, 67.0% (95%CI 59.7-73.6) were in school, 67.8% (95%CI 60.5-74.3) did not work, and 50.0% (95%CI 42.2-57.7) were children of mothers who had not completed high school. The majority (64.6%; 95%CI 57.2-71.4) had never been tested for HIV before the study. Moreover, 38.0% (95%CI 31.1-45.5), 43.6% (95%CI 36.5-50.9), and 23.8% (95%CI 17.8-31.0) reported ICU, MSP, and SDU, respectively (Table 1).

**Table 1.** Sociodemographic and behavioral characteristics of adolescent men who have sex with men aged 15 to 19 years in Fortaleza and São Paulo.

Sociodemographic characteristics	%	95%CI
Age (n=287)		
15-17 years old	46.1	39.0-53.3
18-19 years old	53.9	46.7-61.0
Race/Skin color (n=287)		
White	28.1	22.0-35.1
Black/Brown/Indigenous/Yellow	71.9	64.9-78.0
Currently enrolled in school (n=274)		
No	33.0	26.4-40.3
Yes	67.0	59.7-73.6
Education (n=274)		
Complete or incomplete elementary school	10.2	6.4-15.8
Incomplete high school	27.6	21.6-34.6
Complete high school	62.2	54.8-69.1
Mother’s education (n=243)		
Incomplete high school	50.0	42.2-57.7
Complete high school	50.0	42.3-57.8
Employed (n=275)		
No	67.8	60.5-74.3
Yes	32.2	25.7-39.5
**Behavioral characteristics**		
Have been tested for HIV (n=274)		
No	64.6	57.2-71.4
Yes	35.4	28.6-42.8
Inconsistent condom use (n=274)		
No	62.0	54.5-68.9
Yes	38.0	31.1-45.5
Multiple sexual partners (n=287)		
No	56.4	49.1-63.5
Yes	43.6	36.5-50.9
Sexualized drug use (n=274)		
No	76.2	69.0-82.2
Yes	23.8	17.8-31.0

95%CI: 95% Confidence Interval.

ICU was more frequent among adolescents who had a steady partner aged 20 or over (92.2%; 95%CI 80.2-97.2), who had come out to everyone or almost everyone (64.4%; 95%CI 56.9-71.3), who consumed alcohol weekly (77.3%; 95%CI 66.6-85.4), experienced physical, psychological, and/or sexual violence (71.5%; 95%CI 60.7-80.2), and did not know the serological status of their partners (76.3%; 95%CI 66.5-84.0) (Table 2).

**Table 2.** Factors associated with inconsistent condom use, multiple sexual partners, and sexualized drug use among adolescent men who have sex with men aged 15 to 19 years in Fortaleza and São Paulo.

	Inconsistent condom use		Multiple sexual partners		Sexualized drug use	
	% (95%)	p-value	% (95%CI)	p-value	% (95%CI)	p-value
Sociodemographic characteristics						
Race/Skin color (n=287)						
White	73.1 (57.7-84.4)	0.08	45.8 (32.6-59.7)	0.70	30.0 (18.1-45.5)	0.26
Black/Brown/Indigenous/Yellow	57.7 (49.6-65.9)		42.7 (34.5-51.3)		21.4 (15.0-29.7)	
Education (n=271)						
Complete or incomplete elementary school	52.9 (29.5-75.1)	0.68	33.9 (16.4-57.1)	0.53	19.5 (7.7-41.4)	0.38
Incomplete high school	60.8 (46.5-73.5)		48.3 (35.0-61.8)		17.9 (9.1-31.8)	
Complete high school	63.8 (54.5-72.2)		45.0 (35.8-54.5)		27.3 (19.4-37.1)	
Religion (n=273)						
Protestant Christian	64.2 (46.1-78.9)	0.80	46.6 (30.2-63.9)	0.92	17.1 (7.2-35.7)	0.03
Catholic Christian	56.1 (36.9-73.6)		41.2 (24.1-60.7)		6.0 (2.1-16.0)	
None/Not Christian	62.6 (53.6-70.8)		45.2 (36.5-54.5)		27.2 (19.8-36.1)	
Age of steady partner (n=274)						
No steady partner	46.5 (36.9-56.3)	<0.01	41.8 (32.4-51.8)	0.13	20.1 (13.0-29.8)	0.14
< 20 years	84.3 (72.6-91.6)		57.5 (43.4-70.4)		34.8 (22.0-50.4)	
≥ 20 years	92.2 (80.2-97.2)		38.1 (23.7-54.8)		22.1 (11.8-37.7)	
Came out to everyone or almost everyone (n=275)						
No	10.3 (2.4-35.1)	<0.01	40.5 (11.0-78.9)	0.84	4.23 (0.8-18.8)	<0.01
Yes	64.4 (56.9-71.3)		44.8 (37.5-52.3)		24.7 (18.5-32.2)	
**Sexual behaviors and contextual vulnerabilities**						
Experienced physical, psychological, and/or sexual violence (n=287)						
No	52.9 (42.9-62.6)	0.01	40.0 (31.1-49.7)	0.31	11.7 (7.1-18.6)	<0.01
Yes	71.5 (60.7-80.2)		47.5 (36.7-58.5)		36.2 (26.1-47.8)	
Alcohol consumption in the last 3 months (n=271)						
Never	27.0 (11.5-51.3)	<0.01	30.1 (13.3-54.7)	0.06	-	-
Monthly	57.2 (47.3-66.6)		39.8 (30.9-49.4)		-	
Weekly	77.3 (66.6-85.4)		55.6 (42.7-67.7)		-	
Received money in exchange for sex (n=273)						
No	60.2 (52.0-67.8)	0.15	41.3 (33.7-49.3)	0.01	20.1 (14.4-27.4)	<0.01
Yes	74.1 (56.0-86.5)		66.3 (47.7-81.0)		46.3 (27.5-66.2)	
Sexual intercourse with partners of unknown HIV serological status (n=287)						
No	48.4 (38.5-58.4)	<0.01	71.8 (62.6-79.4)	<0.01	16.4 (10.2-25.4)	0.02
Yes	76.3 (66.5-84.0)		39.3 (29.5-50.1)		31.7 (22.1-43.1)	
**Programmatic and healthcare service factors**						
Current use of pre-exposure prophylaxis (n=287)						
No	61.3 (53.7-68.4)	0.07	43.1 (36.0-50.6)	0.25	23.1 (17.1-30.5)	0.95
Yes	90.3 (52.7-98.7)		64.1 (28.6-88.8)		52.5 (20.3-82.7)	
Used post-exposure prophylaxis (n=274)						
No	61.4 (53.9-68.4)	0.12	45.1 (37.8-52.6)	0.37	24.4 (18.1-32.1)	0.07
Yes	100 (-)		23.1 (2.96-74.7)		23.1 (2.96-74.7)	
Source of care when sick (n=275)						
Family/friends/pharmacy/Nowhere	58.6 (47.2-69.1)	0.12	43.8 (32.9-55.2)	0.72	18.0 (10.7-28.8)	0.25
Public health service	61.3 (50.5-71.0)		43.7 (33.9-54.1)		26.2 (17.9-36.5)	
Private health service	85.5 (65.0-95.0)		55.3 (29.5-78.5)		38.4 (15.2-68.5)	
Participation in NGO activities (n=274)						
No	61.3 (53.5-68.6)	0.56	43.4 (35.9-51.2)	0.28	22.0 (15.9-29.5)	0.05
Yes	68.5 (43.8-85.9)		57.0 (33.8-77.6)		44.0 (22.5-68.0)	

95%CI: 95% Confidence Interval; NGO: non-governmental organization.

The most frequent factors among those who reported MSP were consuming alcohol weekly (55.6%; 95%CI 42.7-67.7), receiving money in exchange for sex (66.3%; 95%CI 47.7-81), and not knowing the serological status of their partners (71.8%; 95%CI 62.6-79.4) (Table 2).

With regard to SDU, it was possible to identify a higher frequency among adolescents who had come out to everyone or almost everyone about their sexual orientation (24.7%; 95%CI 18.5-32.2); suffered physical, psychological, and/or sexual violence (36.2%; 95%CI 26.1-47.8), received money in exchange for sex (46.3%; 95%CI 27.5-66.2), and did not know the serological status of their partners (31.7%; 95%CI 22.1-43.1) (Table 2).

In the multivariate model, the following associated variables were identified (Table 3):

1) ICU - not having a steady partner (APR: 0.5; 95%CI: 0.4-6), having come out to everyone or almost everyone (APR: 6.3; 95%CI 1.5-25.5), consuming alcohol weekly (APR: 2.9; 95%CI 1.3-6.2), looking for family/friends/pharmacy (APR: 0.7; 95%CI 0.5-0.9) or the public healthcare service (APR: 0.7; 95%CI 0.6-0.9) when sick; and having sexual intercourse with partners of unknown HIV status (APR: 1.6; 95%CI 1.3-2.0); 2) MSP - receiving money in exchange for sex (APR: 1.6; 95%CI 1.1-2.2), and having intercourse with partners of unknown HIV status (APR: 2.0; 95%CI 1.4-2.8); 3) SDU - Catholic religion (APR: 0.2; 95%CI 0.1-0.6), having a steady partner < 20 years old (APR: 2.0; 95%CI 1.1-4.0), participating in NGO activities (APR: 2.5; 95%CI 1.5-4.4), experiences of violence (APR: 2.7; 95%CI 1.5-4.7), receiving money in exchange for sex (APR: 1.7; 95%CI 1.1-2.8), and having intercourse with partners of unknown HIV status (APR: 2.1; 95%CI 1.3-3.6).

**Figure 1.**
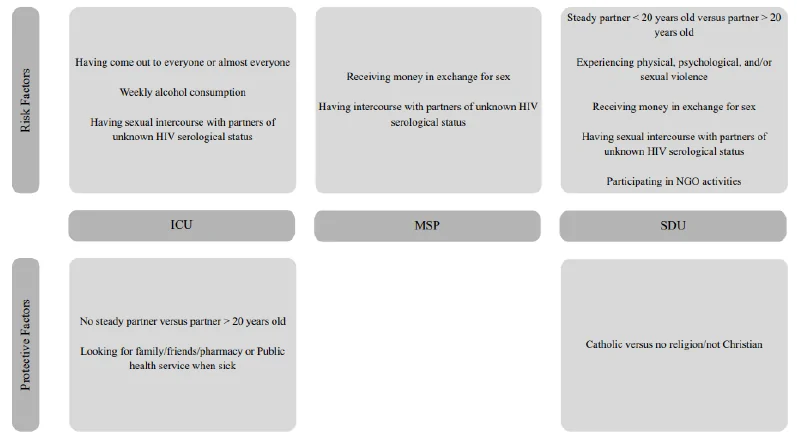
Main risk and protective factors associated with inconsistent condom use, multiple sexual partners, and sexualized drug use among adolescent men who have sex with men aged 15 to 19 years in Fortaleza and São Paulo.

ICU: inconsistent condom use; MSP: multiple sexual partners; SDU: sexualized drug use; NGO: non-governmental organization.

Figure 1 summarizes the main risk and protective factors associated with each outcome, providing a visual overview complementary to the multivariable results shown in Table 2.

**Table 3.** Crude and adjusted prevalence ratios for inconsistent condom use, multiple sexual partners, and sexualized drug use among adolescent men who have sex with men aged 15 to 19 years in Fortaleza and São Paulo.

	Inconsistent condom use		Multiple sexual partners		Sexualized drug use	
	CPR (95%CI)	APR (95%CI)	CPR (95%CI)	APR (95%CI)	CPR (95%CI)	APR (95%CI)
Race/Skin color (n=287)						
White	1	-	-	-	-	-
Black/Brown/Indigenous/Yellow	0.8 (0.6-1.0)*	-	-	-	-	-
Religion (n=273)						
Protestant	-	-	-	-	0.6 (0.2-1.5)	0.7 (0.3-1.6)
Catholic	-	-	-	-	0.2 (0.1-0.6)***	0.2 (0.1-0.6)***
None/Not Christian					1	1
Age of steady partner (n=274)						
No steady partner	0.5 (0.4-0.6)***	0.5 (0.4-0.7)***	1.1 (0.7-1.8)	-	0.9 (0.4-1.9)	1.5 (0.7-3.2)
< 20 years	0.9 (0.8-1.1)*	0.9 (0.7-1.1)*	1.5 (0.9-2.5)	-	1.6 (0.8-3.2)	2.0 (1.1-4.0)**
≥ 20 years	1	1	1	-	1	1
Came out to everyone or almost everyone (n=275)						
No	1	1	-	-	1	-
Yes	6.3 (1.5-25.5)***	4.6 (1.2-18.5)**	-	-	5.8 (1.2-29.3)**	-
Experienced physical, psychological, and/or sexual violence (n=287)						
No	1	-	-	-	1	1
Yes	1.3 (1.1-1.7)**	-	-	-	3.1 (1.7-5.5)***	2.7 (1.5-4.7)***
Alcohol consumption in the last 3 months (n=271)						
Never	1	1	1	-	-	-
Monthly	2.1 (0.9-4.6)	1.7 (0.9-3.4)*	1.32 (0.61-2.81)	-	-	-
Weekly	2.9 (1.3-6.2)***	2.1 (1.1-4.1)**	1.84 (0.86-3.92)*	-	-	-
Received money in exchange for sex (n=273)						
No	1	-	1	1	1	1
Yes	1.2 (0.9-1.6)*	-	1.6 (1.2-2.2)***	1.6 (1.1-2.2)***	2.3 (1.3-3.9)***	1.7 (1.1-2.8)**
Sexual intercourse with partners of unknown HIV serological status (n=287)						
No	1	1	1	1	1	1
Yes	1.6 (1.3-2.0)***	1.6 (1.3-1.9)***	2.1 (1.5-3.0)***	2.0 (1.4-2.8)***	1.9 (1.1-3.4)**	2.1 (1.3-3.6)***
Current use of pre-exposure prophylaxis (n=287)						
No	1	-	-	-	1	-
Yes	1.4 (1.1-1.8)***	-	-	-	2.3 (1.1-4.8)**	-
Used post-exposure prophylaxis (n=274)						
No	1	-	-	-	-	-
Yes	1.61 (1.42-1.82)***	-	-	-	-	-
Source of care when sick (n=275)						
Family/friends/pharmacy	0.7 (0.5-0.9)***	0.7 (0.5-0.9)**	-	-	-	-
Public health service	0.7 (0.5-0.9)***	0.7 (0.6-0.9)**	-	-	-	-
Private health service	1	1				
Participation in NGO activities (n=274)						
No	-	-	-	-	1	1
Yes	-	-	-	-	2.0 (1.01-3.8)**	2.5 (1.5-4.4)***

NGO: non-governmental organization; CPR: Crude prevalence ratio; APR: Adjusted prevalence ratio. All models were adjusted for age and all variables with p<0.20 in the crude analyses; *p≤0.20; **p≤0.05; ***p≤0.01.

## DISCUSSION

The prevalence of sexual risk behavior among AMSM in this study was high. These high levels of vulnerability among AMSM reflect the influence of multiple relational, behavioral, and structural factors that shape how adolescents navigate their sexual partnerships and prevention practices. In regions with high HIV prevalence, such as Brazil, promoting safe sexual behavior, including consistent condom use and pre-exposure prophylaxis (PrEP), is crucial for reducing the spread of HIV. This is especially important among adolescents and young adults (aged 15-24), who have the highest rates of new infections^25^.

Age-discrepant relationships can introduce power imbalances in which adolescents have less autonomy to negotiate condom use or to question risk behaviors. Evidence from a recent qualitative study among South African youth shows that younger partners often perceive older partners as more experienced or authoritative, which reduces their ability to initiate conversations about HIV prevention or condom use^26^. In these relationships, ICU is frequently framed as a sign of trust, commitment, or emotional closeness^27^, even when the older partner’s HIV status is unknown, reinforcing a form of “blind trust” based on relational dynamics rather than actual risk assessment^28^.

Likewise, having come out to everyone or almost everyone was associated with a higher likelihood of ICU. Disclosure of sexual orientation may lead MSM to integrate earlier into adult sexual networks, where being openly gay can facilitate access to partners but also increase vulnerability, given the greater likelihood of condomless sex within these contexts^29^.

Seeking care from community sources (family members, friends, and pharmacies) was associated with a lower likelihood of ICU, suggesting that adolescents who have supportive social networks may benefit from informal guidance and greater access to health information.

Experience of SDU was associated with a higher chance of experiencing physical, psychological, and/or sexual violence among AMSM. A study using latent class analysis identified a syndemic of victimization, depression, and substance use among AMSM^30^. The authors explain that experiences of victimization can worsen depression and lead to maladaptive coping mechanisms, including substance use. Since each of these factors is linked to sexual risk, AMSM affected by all of these factors are particularly vulnerable to engaging in risky sexual behavior, increasing their risk of acquiring HIV and other sexually transmitted diseases (STIs)^30^. Substance use was also associated with exchanging sex for money^31^ ^,^ ^32^. Male sex workers were twice as likely to use illicit drugs or experience physical violence compared to men who are not sex workers^33^.

The association between participation in NGO activities and higher prevalence of SDU in our sample may reflect a complex interplay between vulnerability, health-seeking behaviors, and the role of community-based organizations in HIV prevention and care. Individuals who engage in NGO activities often represent groups with higher baseline vulnerability, including those with multiple risk factors, social disadvantage, or prior exposure to stigma and marginalization. In this sense, our findings may not imply that NGO participation causes risk, but rather that NGOs tend to reach the most vulnerable adolescents, who seek support, information, or prevention and, at the same time, may already engage in higher risk behaviors (e.g., drug use, MSP). Thus, the positive association likely reflects a selection effect (or reverse causality) rather than a causal effect of NGO participation on SDU. This highlights the importance of interpreting this variable as a marker of vulnerability and asserts the need to strengthen NGO-based interventions to combine outreach and prevention with harm-reduction and structural support, especially for AMSM. Evidence from modeling studies in Eastern Europe indicates that MSM-targeted NGOs contribute substantially to reducing HIV incidence by providing prevention services such as condom distribution, testing, and access to care, and are highly cost-effective when maintained long-term^34^.

This study has some limitations. The calculated sample size was not reached in Fortaleza. Although a larger number of seeds was selected for Fortaleza (10) compared with São Paulo (7), this strategy was insufficient to achieve the calculated sample size. This indicates that the limitation was not merely the number of seeds, but rather the reduced network density and lower connectivity among AMSM in Fortaleza during and after the most restrictive phases of the COVID-19 pandemic. The two cities chosen and social networking in both cities were greatly affected by the COVID-19 pandemic. Fortaleza responded by closing schools^35^ and other institutions and public places and discouraging non-essential movement^36^. São Paulo did not pursue active social distancing but did initiate vaccination in January 2021, the first location in Brazil.

A second feature of the different social context in the two cities was that the reported social network size of peers was smaller in Fortaleza than in São Paulo. Youth in Fortaleza commented that they had few opportunities to meet peer MSM, and that most of their contacts were via social media, where they encountered older men who were not included in our study. We believe that the closing of institutions and other issues affecting public circulation compromised networking with peers, and impoverished their personal networks during and following the worst moments of the pandemic, critically affecting the chain-link recruitment process. This prevented the achievement of the target sample size. These factors likely hindered the formation of long recruitment chains, illustrating how contextual and structural constraints can substantially affect RDS performance even when methodological parameters (such as number of seeds) are adequate.

Given the considerations above, we decided to analyze the data from the two cities jointly. This approach offered important advantages within an RDS framework. Because RDS estimators depend on preserving the full recruitment structure and on participants’ network visibility, conducting separate analyses by city would fragment the recruitment chains and produce unstable or biased estimates, particularly in Fortaleza, where chains were shorter, and the sample size was smaller. Pooling the data allowed us to apply the RDS weights appropriately and improved the precision of the estimates. On the other hand, analyzing the two sites together may mask contextual differences between Fortaleza and São Paulo, especially regarding sociocultural factors influencing AMSM vulnerability. These trade-offs should be considered when interpreting the results.

Given our findings on the sexual behaviors of AMSM, it is no surprise that recent studies have found HIV prevalence among AMSM to be about three times higher than that of Brazilian male adolescents drawn from the general population^37^ ^,^ ^38^. To respond to this glaring disparity in a world with test and treat, and elimination targets, it is necessary to address the underlying determinants of HIV infection with strategies targeted to this very specific audience and the issues surrounding overcoming barriers. Targeted access to prevention, such as condoms, PrEP, and harm reduction for substance use, all seem critical to disrupt HIV persistence in key populations and provide truly universal healthcare.

Recommendations for public health responses must consider the distinct contexts of the two cities where the study was conducted. In Fortaleza, the reduced density of peer networks, the predominance of online interactions with older partners, and the stronger impact of pandemic-related restrictions highlight the need for strategies that strengthen community-based outreach and rebuild social support spaces for adolescents. In São Paulo, although social networks appeared broader, challenges include navigating a large and fragmented urban environment, where access to youth-friendly sexual health services is uneven. These contextual differences point to important implications for the Brazilian Unified Healthcare System (SUS). Strengthening accessible, confidential, and adolescent-centered services; expanding prevention initiatives such as PrEP and regular HIV testing; and integrating schools, primary care, and community organizations are essential steps. Addressing structural barriers, such as stigma, fear of disclosure, and limited engagement with healthcare services, remains critical for ensuring that the SUS can adequately respond to the needs of AMSM across diverse settings.

In conclusion, this study provides important insights into the factors associated with sexual risk behaviors among AMSM recruited through RDS in two Brazilian cities marked by distinct social and structural contexts. Despite contextual differences between Fortaleza and São Paulo, we identified consistent patterns of vulnerability associated with peer network limitations, age-discordant partnerships, SDU, and challenges in accessing youth-friendly sexual health services. The findings underscore the need for targeted, adolescent-centered strategies that address both behavioral risks and the broader social conditions shaping AMSM vulnerability. Strengthening programmatic responses within the SUS, expanding prevention initiatives, and improving engagement with community and school-based structures remain essential for reducing HIV risk and supporting the health and well-being of AMSM across diverse urban settings.
